# Ongoing data from the breast cancer prevention trials: opportunity for breast cancer risk reduction

**DOI:** 10.1186/s12916-015-0300-0

**Published:** 2015-03-26

**Authors:** Victor G Vogel

**Affiliations:** Director, Breast Medical Oncology/Research, Geisinger Health System, Danville, PA 17822 USA

**Keywords:** Aromatase inhibitors, Breast cancer risk, Chemoprevention, Risk reduction, Selective estrogen receptor modulators, Tamoxifen

## Abstract

Selective estrogen receptor modulators (SERMs) reduce the risk of recurrence of invasive breast cancer and the incidence of first breast cancers in women who are at increased risk. Multiple, randomized clinical trials have shown both the efficacy and safety of SERMs in reducing the risk of breast cancer. Long-term follow-up as long as 20 years in the randomized trials shows persistent efficacy with acceptable safety. Hormone replacement therapy given concurrently with tamoxifen abrogates its preventive effect, but women with atypical hyperplasia derive particular benefit from SERM therapy. Aromatase inhibitors also reduce the risk of developing invasive breast cancer, but the experience with them for risk reduction is limited to few trials. National organizations have made recommendations to use SERMs and aromatase inhibitors to reduce the risk of breast cancer in high-risk women and additional efforts should be made to increase their use in clinical practice, where the number of women needed to treat to prevent one case of breast cancer conforms to accepted standards of preventive medicine.

## Background

Breast cancer is due, in part, to the stimulation of initiated cancer cells by estrogen through the estrogen receptor (ER). Selective estrogen receptor modulators (SERMs) are competitive inhibitors of estrogen at the receptor and have been used effectively for decades to treat both early and advanced breast cancer. Aromatase inhibitors block the production of estrogen in postmenopausal women and are as effective as SERMs in treating both early and advanced breast cancer. Risk factors, such as family history and benign breast disease, identify women who are at increased risk for developing breast cancer. Investigators have been conducting clinical trials with SERMs and aromatase inhibitors for more than 20 years [1-16]. Cuzick et al. [6] conducted a meta-analysis based on individual-level data from nine randomized trials that compared SERMs with placebo or another drug in women without breast cancer. The meta-analysis included 83,399 women with 306,617 collective years of follow-up and eight of the analyzed trials were placebo-controlled trials, whereas one compared tamoxifen with raloxifene. Overall, there was a 38% reduction in breast cancer incidence, with 42 women needing to be treated to prevent one case of breast cancer, over a 10-year follow-up period; the largest risk reduction was observed in the first 5 years. There was also a significant 73% increase in the incidence of thromboembolic disease with all SERMs and a significant 34% reduction in the incidence of non-vertebral fractures, although information about absolute risks was not available [6]. Long-term follow-up is a useful expansion of the initial reports that guides clinicians in weighing the risks and benefits of a preventive risk reduction intervention.

## The IBIS-I trial

The IBIS-I trial was one of several prospective trials of a SERM to reduce the risk of breast cancer in high-risk women. It was a randomized, controlled trial conducted in both premenopausal and postmenopausal women aged 35 to 70, randomly assigned to receive oral tamoxifen 20 mg daily or matching placebo for 5 years [17]. After a median follow-up of 16 years, 251 breast cancers occurred in 3,579 patients in the tamoxifen group compared with 350 breast cancers in 3,575 women in the placebo group, representing a 29% reduction in risk. The risk of developing breast cancer was similar between years 0 to 10 and after 10 years (31%). The greatest reduction in risk was seen in invasive ER-positive breast cancer (34%) and ductal carcinoma in situ (35%), but no effect was noted for invasive ER-negative breast cancer.

Importantly, 40% of trial participants in IBIS-I used menopausal hormone replacement therapy (HRT) at some time during the treatment phase of the trial. Of potential interest was a non-statistically significant interaction between HRT use and tamoxifen that was reported in the initial results from IBIS-I [8]. Among women who never used HRT or who used it only before the trial, there was a statistically significant reduction in ER-positive breast cancers in the tamoxifen arm compared with the placebo arm (51%). However, for women who used HRT at some stage during the trial, no clear effect of tamoxifen was seen, either overall or for ER-positive tumors. Results were similar regardless of the HRT preparations used, i.e., estrogen only or combined estrogen and progestin. HRT use was not associated with the development of ER-negative breast cancers, either during the active treatment period or the subsequent period.

## Discussion

While the IBIS-I trial data show inhibition of the benefit of tamoxifen therapy among women taking oral HRT, it is known that conjugated equine estrogen treatment alone without a progestin does not increase the incidence of invasive of breast cancer. In fact, published data show that conjugated equine estrogen alone reduces the risk of invasive breast cancer in postmenopausal women [18]. We are not given complete information about what HRT preparations were used by the women in the trial. The use of HRT may explain, at least in part, why the reduction of breast cancer risk in IBIS-I (28% reduction in years 0 to10) was less than that seen in the other reported risk reduction trials using tamoxifen. The use of conjugated estrogens alone in IBIS-I may have nevertheless partially abrogated the potential benefit of tamoxifen.

Given the results from other published risk reduction trials, it is somewhat surprising that the risk reduction during the first 10 years of follow-up in IBIS-I was only 29% and increased to just 31% in subsequent years. It is reassuring that there is a persistent and enduring effect of tamoxifen: even after 20 years of follow-up, the estimated risk of developing all types of breast cancer was 12.3% in the placebo group compared with only 7.8% in the tamoxifen group. These data indicate that the number needed to treat with 5 years of tamoxifen to prevent one breast cancer in the next 20 years was only 22 women. More importantly, the risk reduction for ER-positive cancers was greater, but the number needed to treat and prevent one case of ER-positive breast cancer was 29 women.

As in other SERM risk reduction trials [19], there was a significant reduction in ductal carcinoma in situ, which was 45% during 0 to 10 years of follow-up but decreased to only 9% with 10 or more years of follow-up. The reduction in the risk for ER-positive cancers during 10 years was 32%, and increased to 37% among women with 10 or more years of follow-up.

There were more ER-negative breast cancers in the tamoxifen group of IBIS-I after 10 years of follow-up than in the placebo group, although the reasons for this are not obvious. The odds for deep vein thrombosis with tamoxifen were increased by 73%, but this increased risk was observed only during the first 10 years of follow-up. These data are similar to those reported in the Breast Cancer Prevention Trial [1,2] and the STAR trial [10,11].

The non-significant increase in ER-negative tumors after 10 years seen in IBIS-I has been attributed by some observers to a suppression of the appearance of these tumors while tamoxifen therapy was being administered followed by a release of the suppression when tamoxifen therapy ended. This, however, should not be viewed as a failure of tamoxifen therapy. The large reduction in the incidence of ER-positive breast cancers significantly outweighs the small number of ER-negative tumors that occurred in tamoxifen-treated women.

Although the authors of these long-term data from IBIS-I state that it is of concern that a reduced incidence of breast cancer with tamoxifen has not translated into mortality reduction, they reported that only 9.5% of incident breast cancer cases have died. They acknowledge that the power of their analysis for mortality reduction is much lower than that for incidence and note that the observed reduction in incidence should have translated to an estimated 18% reduction in breast cancer mortality, but their statistical power to detect such a reduction in mortality was only 12% given the small number of deaths that occurred. The trial should not be interpreted, therefore, as having failed to show a reduction in mortality.

## Aromatase inhibitors for reducing the risk of breast cancer

SERMs are not the only agents known to reduce the risk of breast cancer in women at increased risk. In the IBIS-II trial, women were randomly assigned to receive anastrozole or placebo [20]. After a median follow-up of 5 years, 2% in the anastrozole group and 4% in the placebo group developed breast cancer (hazard ratio 0.47, 53% reduction in incidence). The predicted cumulative incidence of all breast cancers after 7 years was 5.6% in the placebo group and 2.8% in the anastrozole group. For women who entered the trial with either lobular carcinoma in situ or atypical hyperplasia, the risk reduction in the incidence of invasive breast cancer was 69% after 7 years of therapy. In the MAP3 trial, 65% fewer invasive breast cancers were detected in women given the aromatase inhibitor exemestane compared with those women assigned placebo [12].

## Weighing the risks and benefits of reducing the risk of breast cancer

In order for a preventive strategy to be both effective and efficient, we need an easily identified target population, criteria for identifying those who would benefit from a risk reduction strategy, a safe and effective agent, an informed group of practitioners who can provide care to the high-risk group, and an educated population of patients who understand the advantages and the risks of taking a drug to modify their risk [21].

Freedman et al. [22] developed a benefit/risk index to quantify benefits from chemoprevention with tamoxifen or raloxifene, the SERMS used in the NSABP STAR trial. The benefits and risks of raloxifene and tamoxifen are described in tables that can help identify groups of women for whom the benefits outweigh the risks. The published benefit/risk indices indicate that raloxifene is better than tamoxifen for women aged 50 years or older with a uterus. For women without a uterus, the benefit/risk profile for raloxifene is similar to that for tamoxifen. It is possible for a health care provider to obtain a benefit/risk index from the published tables, and by combining this information with that on clinical features and personal preferences, the provider and patient can make an informed decision.

Despite the compelling results of chemoprevention trials using SERMs for breast cancer risk reduction, there has been minimal use of either tamoxifen or raloxifene by women at risk for breast cancer. A number of reasons have been put forth to explain why patients may not be willing to adopt a SERM for breast cancer risk reduction. HRT is still widely used by postmenopausal women, even after published results showed an associated increased risk for breast cancer, but its use is contraindicated with concurrent SERM therapy. Patients erroneously perceive the risks of SERM therapy to be greater than its benefits, and they perceive the risks of therapy-related side effects to be greater than their risk of breast cancer [16]. This problem is confounded by the fact that they (and perhaps their physicians) are confused by the concept of probabilistic risk. Finally, they fear endometrial cancer out of proportion to its true tamoxifen-related risk and do not understand that there is no increased risk of uterine malignancy associated with raloxifene; we must hope that lasofoxifene does not soon suffer the same fate of misinformation. Additional reasons not to adopt and initiate strategies to reduce the risk of breast cancer include the fear of adverse effects, medication costs, lack of reasonably accurate and feasible methods for assessing personal individual risk, and lack of established risk thresholds that maximize benefit and minimize harms.

## The special case of cellular atypia

A number of studies have indicated that breast cancer risk is increased following detection of atypical hyperplasia, making women with these lesions ideal candidates for breast cancer risk reduction [1,2,7,10-12,17,23,24]. Thus, chemoprevention with a SERM may be particularly beneficial to women with atypical hyperplasia, a 5-year Gail model risk of more than 5%, lobular carcinoma in situ, or two or more first-degree relatives with breast cancer based on the published data reviewed in this chapter. There are no primary prevention studies to evaluate the optimum duration of tamoxifen therapy for reducing the risk of breast cancer, but completed clinical trials in the adjuvant therapy setting show that using tamoxifen for 10 years is more beneficial than only 5 years of use. No trials are being conducted or are planned to examine the ideal duration of therapy in the risk-reduction setting.

## Summary and clinical recommendations

Based on all of the available published data, the American Society of Clinical Oncology (ASCO) recommended, in 2013, that in women at increased risk of breast cancer aged ≥35 years, tamoxifen (20 mg per day for 5 years) should be discussed as an option to reduce the risk of ER-positive breast cancer [25]. In postmenopausal women, raloxifene (60 mg per day for 5 years) and exemestane (25 mg per day for 5 years) should also be discussed as options for breast cancer risk reduction. Those women at increased breast cancer risk are defined as individuals with a 5-year projected absolute risk of breast cancer ≥1.66% (based on the National Cancer Institute Breast Cancer Risk Assessment Tool or an equivalent measure) or women diagnosed with lobular carcinoma in situ. ASCO encouraged health care providers to discuss the option of chemoprevention among women at increased breast cancer risk and urged that the discussion include the specific risks and benefits associated with each chemopreventive agent. Because the risk of clotting increases with age, and because both stroke and pulmonary embolism are potentially life-threatening consequences of tamoxifen therapy, careful consideration must be given to risks versus benefits in older postmenopausal women who are considering tamoxifen for risk reduction.

At a minimum, a risk assessment encounter should include a clear description of the benefits and risks of taking a SERM for the individual woman, including a description of the side effects experienced by published study participants. For example, the counselor should take into account particular risk factors to see if the woman is subject to increased risk of SERM, aromatase inhibitor-induced stroke, or endometrial cancer.

## Abbreviations

ASCO: American Society of Clinical Oncology
ER: Estrogen receptor
HRT: Hormone replacement therapy
SERMs: Selective estrogen receptor modulators
